# Feasibility of intraventricular administration of etoposide in patients with metastatic brain tumours

**DOI:** 10.1054/bjoc.2001.1841

**Published:** 2001-06

**Authors:** G Fleischhack, S Reif, C Hasan, U Jaehde, S Hettmer, U Bode

**Affiliations:** Department of Paediatric Haematology/Oncology, University of Bonn, Adenauerallee 119 Bonn, D-53113; Department of Clinical Pharmacy, Institute of Pharmacy, Free University of Berlin, Kelchstrasse 31, D-12169 Berlin; Department of Clinical Pharmacy, Institute of Pharmacy, University of Bonn, An der Immenburg 4, D-53121 Bonn, Germany

**Keywords:** etoposide, intraventricular therapy, metastatic medulloblastoma, pharmacokinetics

## Abstract

As the systemic administration of etoposide is effective in the treatment of relapsed and metastatic brain tumours, a pilot trial was designed to study the feasibility of intraventricular administration of etoposide in such patients. 14 patients aged 2.1 to 33.2 years were treated with intraventricular etoposide simultaneously with either oral or intravenous chemotherapy with trofosfamide or carboplatin and etoposide. In 59 courses (1–12/patient) 0.5 mg etoposide was administered daily via an indwelling subcutaneous reservoir for 5 consecutive days every 2–5 weeks over a period of 0–11 months. During 15 courses in 5 patients serial CSF samples were obtained and etoposide levels were determined by reversed-phase HPLC. Side effects included transient headache and bacterial meningitis, each during 2 courses. Pharmacokinetic data analysis in the CSF (11 courses, 4 patients) revealed a terminal half-life of 7.4±1.2 hours and an AUC of 25.0 ± 9.5 μg h ml^–1^(mean ± standard deviation). The volume of distribution at steady state and total clearance exhibited a large interindividual variability with mean values of 0.16 l and 0.46 ml min^–1^respectively. Intraventricularly administered etoposide is well tolerated. CSF peak levels exceed more than 100-fold those achieved with intravenous infusions. Further studies should be focused on optimizing the dose and schedule and on determining the effectiveness of intraventricularly administered etoposide. © 2001 Cancer Research Campaign http://www.bjcancer.com


British Journal of Cancer (2001) 84(11), 1453–1459
© 2001 Cancer Research Campaign
doi: 10.1054/ bjoc.2001.1841, available online at http://www.idealibrary.com on IDEAL

### Feasibility of intraventricular administration of etoposide in patients with metastatic brain tumours

**G Fleischhack[1], S Reif [2], C Hasan[1], U Jaehde[3], S Hettmer[1] and U Bode[1]**

[1]Department of Paediatric Haematology/Oncology, University of Bonn, Adenauerallee 119, D-53113 Bonn; [2]Institute of Pharmacy, Department of Clinical Pharmacy, Free University of Berlin, Kelchstrasse 31, D-12169 Berlin; [3]Institute of Pharmacy, Department of Clinical Pharmacy, University of Bonn, An der Immenburg 4, D-53121 Bonn, Germany

**Summary** As the systemic administration of etoposide is effective in the treatment of relapsed and metastatic brain tumours, a pilot trial was designed to study the feasibility of intraventricular administration of etoposide in such patients. 14 patients aged 2.1 to 33.2 years were treated with intraventricular etoposide simultaneously with either oral or intravenous chemotherapy with trofosfamide or carboplatin and etoposide. In 59 courses (1–12/patient) 0.5 mg etoposide was administered daily via an indwelling subcutaneous reservoir for 5 consecutive days every 2–5 weeks over a period of 0–11 months. During 15 courses in 5 patients serial CSF samples were obtained and etoposide levels were determined by reversed-phase HPLC. Side effects included transient headache and bacterial meningitis, each during 2 courses. Pharmacokinetic data analysis in the CSF (11 courses, 4 patients) revealed a terminal half-life of 7.4±1.2 hours and an AUC of 25.0 ± 9.5 µg h $ml^{-1}$ (mean ± standard deviation). The volume of distribution at steady state and total clearance exhibited a large interindividual variability with mean values of 0.16 l and 0.46 ml $min^{-1}$ respectively. Intraventricularly administered etoposide is well tolerated. CSF peak levels exceed more than 100-fold those achieved with intravenous infusions. Further studies should be focused on optimizing the dose and schedule and on determining the effectiveness of intraventricularly administered etoposide. © 2001 Cancer Research Campaign http://www.bjcancer.com

**Keywords**: etoposide; intraventricular therapy; metastatic medulloblastoma; pharmacokinetics

30 to 60% of patients with medulloblastoma and other malignant brain tumours will relapse within 5 years of initial therapy, mostly with a presentation as metastatic disease (Kuehl et al, 1998; Perek et al, 1999). Due to the multimodality of the primary treatment, therapeutic options in relapses are limited. There are few surgical and radiotherapeutic options for local treatment. The number of chemotherapeutic agents capable of passing the blood–brain barrier after systemic administration or suitable for dose escalation or for intrathecal administration is likewise limited. The ‘gold standard’ drugs for intrathecal cytostatic therapy are methotrexate and cytarabine. While both are highly effective in CNS leukaemia/lymphoma, only methotrexate seems to be effective in the treatment of malignant brain tumours (Blaney et al, 1991a, 1991b; Schmandt and Kuehl, 1998). Cytarabine alone has only poorly documented activity in malignant brain tumours, and methotrexate shows an augmented CNS toxicity after CNS irradiation (Rubinstein et al, 1975; Blaney et al, 1991b). Other cytostatic agents tested recently for intra-CSF (cerebrospinal fluid) administration either show rapid clearance from the CSF (thiotepa and ACNU), lack active metabolites (thiotepa) (Blasberg et al, 1975) or have substantial toxicity (mafosfamide) (Slavc et al, 1998). Agents studied in preclinical or clinical phase I/II trials are diaziquone (Berg et al, 1992), depot cytarabine (Chamberlain et al, 1993), topotecan (Blaney et al, 1995), temozolomide (Sampson et al, 1999), FdUrd (5-fluoro-2´-deoxyuridine) (Nakagawa et al, 1999) and monoclonal antiganglioside antibodies (Bergman et al, 1999).

*Received 4 July 2000*
*Revised 20 February 2001*
*Accepted 19 March 2001*

*Correspondence to:* G Fleischhack

Experimental data on medulloblastoma cell lines as well as clinical data on systemic administration of etoposide in patients with neuroectodermal brain tumours reveal a significant cytotoxic activity of etoposide in these tumours (Tomlinson et al, 1991; Dunkel et al, 1998). However, after systemic intravenous or oral administration the penetration of etoposide into the cerebrospinal fluid is extremely poor (less than 3%), with high interindividual variability (Kiya et al, 1992). While concentrations of 0.1 to 10 µg $ml^{-1}$ are cytotoxic in vitro, depending on cell line and exposure time, the cytotoxic levels of etoposide in plasma and CSF are not well known (Henwood and Brogden, 1990).

In 1992 van der Gaast et al reported the successful intra-CSF administration of etoposide in 2 patients with malignant meningeosis resulting from small-cell lung carcinoma and lymphoblastic leukaemia. Etoposide was administered intraventricularly via an ommaya reservoir in 1 patient and intralumbarly in the second patient. One was given in a dose of 0.5 mg etoposide for 5 consecutive days. During a second course 3 weeks later the same dose was applied 10 times every 12 hours. Both patients showed no treatment-related side effects but a clear clinical and cytological response. This was the first report of safe intraventricular administration of low doses of etoposide in humans.

Encouraged by these results we initiated a pilot trial to study the feasibility of intraventricularly administered etoposide in children and adolescents with relapsed metastatic brain tumours.

### PATIENTS AND METHODS

### Patient eligibility

The study was designed as an open comparative multicentre investigation that began in February 1998. The diagnosis of metastatic brain tumour (i.e. medulloblastoma, primitive neuroectodermal tumour, glioblastoma, ependymoma) was confirmed in all patients by histological examination of the primary tumour biopsy or of a tumour biopsy at relapse by a reference neuropathologist according to the WHO classification (Kleihues et al, 1993) and by typical MRI (magnetic resonance imaging) findings. Patients aged above 1 year with refractory or relapsed metastatic malignant brain tumours (i.e. spinal, ventricular and/or parenchymatous metastases and/or meningeosis) were eligible. Patients who were shunt-dependent or displayed a proximal CSF flow obstruction or severe organ insufficiencies (e.g., renal, cardiac, pulmonary or hepatic), life-threatening infections, severe neurologic lesions and/or therapy-resistant seizures (WHO scale 4) were excluded (World Health Organisation, 1979).

### Study design

The study was conducted in accordance with the updated declaration of Helsinki and approved by the local ethics committee of the University of Bonn. Prior to enrolment in the study, all patients and their parents were informed of the investigational nature of the study and the potential risks of this regimen as well as of the poor prognosis of relapsed metastatic brain tumours, and their consent to treatment was obtained. Patient data were collected consecutively, recorded on data sheets prepared centrally beforehand, checked by the principal investigator and deposited in a protected database at the University of Bonn.

### Patient characteristics

Between February 1998 and October 1999 14 patients were enrolled in the trial from departments of paediatric haematology/oncology in Germany. Patient baseline characteristics at study entry are listed in Table 1. Current relapses were documented both by symptoms and by the MRI in 8 patients, only by MRI on routine examination in 6 patients, and additionally by detection of tumour cells in the CSF of 5 patients. At study entry disseminated disease was seen in all patients; 11 patients had multifocal metastases, 5 additional local relapses, 3 meningeosis and 10 spinal or ventricular metastases.

At the time of initial diagnosis all patients were treated with total ($n$ = 11) or subtotal ($n$ = 3) surgical excision of the primary tumour. 12 patients received craniospinal irradiation and 11 were treated with systemic multiagent chemotherapy according to the German protocols HIT'88/89 ($n$ = 2), HIT'91 ($n$ = 8) or HIT-SKK'92 ($n$ = 1) (Kuehl et al, 1993, 1998). At the time of previous relapses 6 patients received systemic chemotherapy and 4 local irradiation. At the time of the current relapses prior to entry into this study 5 patients underwent repeated surgery (2 subtotal resections, 3 biopsies), one was treated with systemic chemotherapy, and another underwent repeated irradiation. Only 1 patient (no. 14) received intraventricular therapy with methotrexate prior to this study.

2 patients (10.2 and 32.5 years old) treated with continuous i.v. infusion of etoposide for a newly diagnosed disseminated medulloblastoma were not given etoposide intraventricularly and served as a second experimental group for comparison of the pharmacokinetic data.

### Chemotherapy

Etoposide was used in the form of Vepesid® (Bristol-Myers Squibb, Germany). A 5 ml vial contained 100 mg etoposide and, as solvents, 150 mg benzyl alcohol, 3250 mg macrogol 300, 1210 mg absolute ethyl alcohol, citric acid and polysorbate 80. For intraventricular administration via an indwelling Ommaya or Rickham reservoir the commercial solution was diluted 200-fold with preservative-free saline, yielding a final concentration of 0.1 mg $ml^{-1}$. The final solution was injected over a 2-minute period after draining of three 2 ml samples of CSF from the reservoir for discarding, for pharmacokinetic analysis and for flushing the reservoir after the injection. 0.5 mg etoposide was administered daily via an Ommaya or Rickham reservoir for 5 consecutive days. The courses were repeated every 2 to 5 weeks (median = 4 weeks) over a period of 0 to 11 months (median = 3.5 months). A total of 59 courses were administered.

Concurrently or immediately after the intraventricular etoposide therapy, all patients were treated with a course of systemic chemotherapy consisting of continuous intravenous (CIV) infusions of carboplatin and etoposide in conventional (800 mg $m^{-2}$ and 400 mg $m^{-2}$ respectively over 96 hours) or high doses (2000 mg $m^{-2}$ and 1000 mg $m^{-2}$ respectively over 96 hours), or orally with etoposide and trofosfamide (25 mg $m^{-2}$ and 100 mg $m^{-2}$ respectively for 21 days) repeated every 4 to 5 weeks. Antiemetics and analgesics were rarely necessary as supportive medication.

Both patients of the second experimental group received a CIV infusion of carboplatin and etoposide in a dose of 800 mg $m^{-2}$ and 400 mg $m^{-2}$ respectively over 96 hours and concurrently 8 times 1 mg methotrexate every 12 hours intraventricularly via an Ommaya reservoir.

### Analysis

### Toxicity and response

Haematological and non-haematological toxicity was assessed in all patients according to the WHO grading system (World Health Organization, 1979).

The tumour response was evaluated as the best overall response and assessed by clinical symptoms, CSF cytology and MRI. The radiographic response on MRI, i.e. the increase, decrease or resolution of the leptomeningeal, ependymal or parenchymal enhancement, and tumour size, were noted. Complete remission (CR) was defined as complete disappearance of all tumour sites, (ii) partial remission (PR) as a decrease of more than 50% in all tumour sites, (iii) stable disease (SD) as less than 25% increase or less than 50% decrease in tumour sites and no appearance of a new tumour site, (iv) a mixed response (MR) as concurrence of partial response in one or more tumour sites and progression of another tumour site or manifestation of a new tumour, and (v) progressive disease (PD) as an increase of more than 25% in one or more tumour sites and/or appearance of new tumours or the onset of meningeosis.

The duration of progression-free survival (PFS) was calculated from initiation of the intraventricular therapy with etoposide to documented progression or relapse, to death by any cause, or to the last day of follow-up.

### Pharmacokinetic analysis

### Sampling

In the main experimental group, CSF samples were taken daily immediately before intraventricular etoposide administration (trough levels, during 15 courses in 5 patients) and 15 minutes after etoposide administration (peak levels, during 11 courses

© 2001 Cancer Research Campaign in 4 patients). On day 1 (during 11 courses in 4 patients) serial samples were collected 0.25, 1, 2, 4, 8, 12 and 24 hours after etoposide injection. Blood samples were taken simultaneously 2 hours (4 courses) or 4 hours (3 courses) after intraventricular administration during courses without systemic etoposide therapy. CSF levels of intralumbar etoposide were not recorded because lumbar spinal metastases were present in 4 of 5 patients studied. In 2 of these 4 patients a scintigraphic CSF flow study was performed and showed partial obstruction in the lumbar spinal canal but no obstruction in the more proximal regions. In the other 3 patients, whose ventricular CSF samples were used for calculation of pharmacokinetic parameters, an undisturbed CSF flow was expected in view of the results of the pre-treatment cerebral and spinal MRI.

In the second experimental group, serial CSF samples for etoposide analysis were collected during 5 courses before infusion, at the steady state of continuous infusion after 24, 48, 72 and 96 hours, and 24 hours after the end of infusion.

CSF and blood samples were collected in heparinized tubes (heparin ammonium), rapidly centrifuged at 10 500 ***g*** for 2 minutes within 20 minutes of collection, and frozen at –20˚C pending analysis.

### *Assay methodology*

Etoposide levels in the CSF were assayed by reversed-phase high-performance liquid chromatography (HPLC) using two previously described methods (Farina et al, 1981; Stremetzne et al, 1997). In brief, a Hypersil® ODS RP-18 column (Knauer, Berlin, Germany) was used as the stationary phase. The mobile phase consisted of methanol/0.01 M $Na_2HPO_4$ (45:55 v/v) and was adjusted with $H_3PO_4$ to pH 6.0. CSF samples (50 µl) were diluted with 25 µl 0.02 M $Na_2HPO_4$ (pH 5.3) in order to prevent etoposide degradation during chromatographic analysis.

Plasma samples (100 µl) were mixed with 250 µl acetonitrile for precipitation of plasma protein and centrifuged at 3200 ***g*** for 10 minutes. The supernatant was evaporated to dryness and the residue was dissolved in 100 µl of mobile phase. 40 µl of the respective mixtures were injected onto the HPLC system. The flow rate was set to 0.4 ml $min^{-1}$ and etoposide was quantified for CSF analysis by electrochemical detection and for plasma analysis by UV detection (210 nm). At electrochemical detection the potentials of the dual electrode cell were set to 100 mV and 500 mV respectively. The limit of quantification of both methods was 0.01 µg $ml^{-1}$. Accuracy and precision met the international criteria for the validation of bioanalytical methods.

### *Pharmacokinetics*

Pharmacokinetic data analysis was performed using the SIPHAR Win software package (Simed, France). A 2-compartment model was fitted to individual CSF concentration time profiles in order to estimate pharmacokinetic parameters such as clearance ($Cl_{tot}$), half-lives ($t_{1/2\ alpha}$, $t_{1/2\ beta}$), the volume of distribution at steady state ($V_{ss}$) and the area under the curve (AUC).

### Statistical analysis

The data were analysed using descriptive statistics to evaluate pre-treatment and post-treatment patient characteristics as well as progression-free survival (PFS). The interindividual and intraindividual variability of the pharmacokinetic parameters was calculated on the basis of the mean and the standard deviation of each patient. The Mann–Whitney U test was used for comparison of the pharmacokinetic parameters in the different groups. In all statistical tests values of $P < 0.05$ at alpha < 0.05 were considered significant. Statistical calculations were performed with the SPSS software package (Statistical Program for Social Science, version 9.0.1., Chicago, USA).

### RESULTS

### Toxicity

All 59 courses of intraventricular etoposide were evaluable for toxicity. Each patient received a median of 4 courses (range: 1 to 12 courses). Nonhaematological side effects were observed in the form of mild transient headache in 2 courses and of infectious complications as meningitis in 2 of 59 courses. Both were treated successfully with analgesics and antibiotics respectively. Secondary to a malfunction, one patient required reservoir revision following the fourth course of intraventricular etoposide. One patient developed recurrent headache and vomiting about 12 hours after the first administration of intraventricular etoposide. Prior to treatment he had craniospinal meningeosis documented by MRI accompanied by a high ventricular CSF protein level (1266 mg $1^{-1}$) and CSF cytology positive for tumour cells. Therapy with steroids, antiemetics and analgesics was intensified and a second intraventricular administration of etoposide was performed 24 hours after the first application. About 4 hours after the second administration the patient presented with headache, vomiting, temporary confusion and transient coma. One day later the patient had a generalized seizure associated with a hyponatraemia resulting from increased intracranial pressure and inappropriate secretion of the antidiuretic hormone. At this time an MRI scan showed rapid progression of disease with marked meningeosis and a beginning brain oedema. After antiepileptic therapy the seizure stopped immediately, and after diuretic and steroid therapy the coma subsided within 24 hours. The patient received no further chemotherapy and died from progressive disease one month later.

Apart from these transient side effects, no acute or chronic toxic encephalopathy, no pleocytosis or aseptic meningitis, and no toxic death were observed. There was no haematological toxicity attributable to intraventricular etoposide.

### Response

The effectiveness of the intraventricular therapy could not be assessed independently in all patients because 13 patients received systemic chemotherapy simultaneously and one underwent irradiation immediately before the intraventricular etoposide (Table 1).

Clinically, 5 patients showed a clear improvement in neurological symptoms or pain reduction, 6 showed no changes, and 3 had progressive symptoms.

Immediately before treatment, CSF cytology was positive for tumour cells in 5 patients, in 3 cases exclusively in the lumbar CSF (once in 1 patient, twice in 2 patients) and in 2 cases in the ventricular CSF (both twice). CSF cytology controlled by repeated ventricular and lumbar CSF samples was evaluable in only 4 of these 5 patients (nos. 1, 4, 5 and 7). These 4 patients had clearance of their CSF following 1, 2 and 5 courses of intraventricular etoposide respectively. Negative CSF cytology was documented for the next

*© 2001 Cancer Research Campaign*

**Table 1** Patient characteristics and outcome

| Patient/ Gender/ Tumour/ No. of Relapse | Age at relapse/ Time from Dx to Current relapse (years) | Relapse Diagnosis | Relapse therapy besides IVC etoposide | | | IVC etoposide | | CSF cytology prior to after IVC etoposide (*n*) | | Best response MRI and CSF cytology |
|---|---|---|---|---|---|---|---|---|---|---|
| | | | Surgery | Chemotherapy (*n*) | Irradiation | Courses (*n*) | Duration (months) | | | |
| 1/ m /MB/2 | 17 / 7.9 | M2+M3+LR | prior R2 | concurrent[1](6) | no | 9 | 5.5 | pos | neg (5) | PR |
| 2/ f /MB/3 | 25 / 11.6 | M3+LR | prior R1 | concurrent[1] (3)[2](9) | no | 12 | 11 | neg | neg | PR |
| 3/ m /MB/1 | 12 / 2.2 | M2+LR | no | concurrent[1](4)[3](1) | after | 5 | 5 | neg | neg | PR |
| 4/ f /MB/1 | 5 / 0.9 | M3+LR+MM | no | concurrent[1](4)<br>after[2](2) | no | 4 | 4 | pos | neg(2)<br>pos(4) | MR |
| 5/ m /MB/1 | 10 / 3.7 | M2 | prior R2 | concurrent[1](3)<br>after[2] (6) | after | 3 | 2 | pos | neg(1) | CR |
| 6/ m /MB/1 | 5 / 1.0 | M2 | no | concurrent[1](3) | no | 3 | 3.5 | neg | neg | PR |
| 7/ f /MB/2 | 33 / 26.8 | M3 | no | concurrent[1](2) | no | 2 | 2 | pos | neg(2) | PR |
| 8/ m /MB/2 | 12 / 4.3 | M2 | prior R1 | concurrent[1](6) | after | 6 | 7 | neg | neg | CR |
| 9/ m /MB/1 | 13 / 1.2 | M2 | no | prior[1](4)<br>concurrent[1](4)<br>after[2](11) | no | 4 | 3.5 | neg | neg | MR |
| 10/ m /MB/1 | 8 / 1.0 | M3+MM | no | concurrent[3](1) | no | 1 | 0 | pos | n.e. | PD |
| 11/ m /MB/2 | 23 / 11 | M3+MM | no | no | prior | 5 | 4 | neg | neg | PR |
| 12/ m /MB/1 | 11 / 2.0 | M3 | prior R2 | concurrent[1](2) | no | 2 | 2 | neg | neg | PR |
| 13/ f /EP/4 | 32 / 2.8 | M3+LR | no | concurrent[1](2) | no | 2 | 2 | neg | neg | SD |
| 14/ m /GB/1 | 2 / 0.8 | M3 | no | concurrent[1](1) | no | 1 | 1 | neg | neg | PD |

m = male; f = female; MB = medulloblastoma; EP = ependymoma; GB = glioblastoma; No. = number; Dx = diagnosis; M2 = ventricular, parenchymal or ependymal metastases in the brain; M3 = spinal metastases; LR = local relapse, MM = malignant meningeosis; IVC = intraventricular; R1 = subtotal resection; R2 = biopsy; [1]conventional dose of carboplatin and etoposide intravenously; [2]conventional dose of etoposide and trofosfamide orally; [3]high dose of carboplatin and etoposide intravenously; pos = positive; neg = negative; n.e. = not evaluable; MRI = magnetic resonance imaging; *n* = number of courses.

*© 2001 Cancer Research Campaign*

2 (nos. 4, 5 and 7) and 4 (no. 1) courses of intraventricular etoposide. Later, 2 of them (nos. 4 and 7) developed CSF dissemination during systemic and intraventricular chemotherapy, while the other 2 showed negative CSF cytology throughout the observation time. 9 patients with initially negative CSF cytology remained negative throughout the treatment period (1 to 11 months).

With respect to the concurrent administration of intraventricular and systemic chemotherapy, the best responses documented by both MRI and CSF cytology were 2 CR, 7 PR, one SD, 2 MR and 2 PD following 1 to 4 months of study treatment.

In 2 patients (nos 1 and 2) with spinal metastases, 111-Indium-DTPA scintigraphy and somatostatin receptor scintigraphy performed simultaneously with the MRI scan showed a reduction in spinal CSF flow obstruction and a marked tumour regression. 6 of 10 patients with spinal or ventricular metastases at the time of the current relapse showed initial regression (CR, PR) of these metastases in the MRI scan. However, 2 of these 6 patients (nos 4 and 7) developed new parenchymatous cerebral tumour lesions during the continued systemic and intraventricular therapy.

### Pharmacokinetic findings

The pharmacokinetic analysis was performed during 11 courses of intraventricular etoposide in 4 patients. Figure 1 shows the CSF concentration time profile (mean, standard deviation) following intraventricular etoposide administration on the first day of each course for all analysed patients. The ventricular etoposide concentration decreased in a similar biexponential fashion. The highest concentrations were measured 0.25 hours after intraventricular etoposide administration. Following administration, significant cytotoxic levels of total etoposide above 1 µg ml$^{-1}$ and 0.1 µg ml$^{-1}$ were detected for 4 hours and 24 hours respectively (Table 2).

In the group receiving no systemic etoposide therapy, plasma levels of etoposide were not detectable 2 (4 courses) and 4 hours (3 courses) respectively after intraventricular etoposide administration.

The pharmacokinetic data listed in Table 3 were determined using a 2-compartment model for all courses. With the exception of the volume of distribution ($P = 0.042$), no significant intergroup differences were detected. Independent of concurrent systemic etoposide therapy, the interindividual variability of the pharmacokinetic parameters was more than 3 times higher than the intraindividual variability (i.e. AUC 67.9% vs. 13.8–19.4%, CI 81.1% vs. 15.9–19.8%, $V_{ss}$ 107.0% vs. 21.7–25.0%).

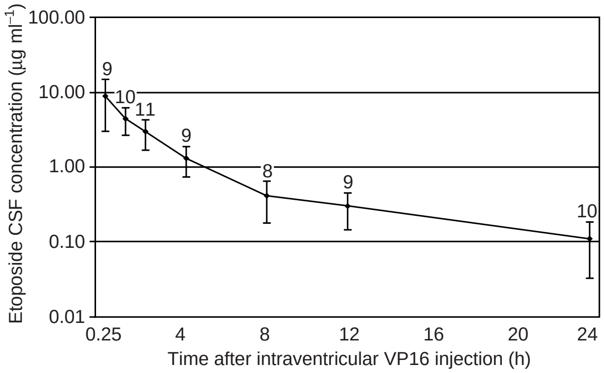

**Figure 1** CSF concentration time profile following intraventricular etoposide administration (0.5 mg) on the first day of a 5-day schedule (mean ± standard deviation, in whole 11 courses in 4 patients, in part the number of measurements is given above each data point)

**Table 2** CSF concentrations of etoposide following the first day of intraventricular administration of 0.5 mg etoposide

| Time after administration (hours) | Number of courses[1] (*n*) | Etoposide concentration (mean ± SD) (µg ml$^{-1}$) |
|---|---|---|
| 0.25 | 9 | 9.03 ± 5.98 |
| 1.00 | 10 | 4.49 ± 1.79 |
| 2.00 | 11 | 3.03 ± 1.31 |
| 4.00 | 9 | 1.31 ± 0.56 |
| 8.00 | 8 | 0.42 ± 0.24 |
| 12.00 | 9 | 0.30 ± 0.16 |
| 24.00 | 10 | 0.11 ± 0.08 |

[1]Etoposide levels were measured during 11 courses in four patients (nos. 1, 2, 3, 13). During 9 courses in 4 patients both levels at 0.25 and 24 hours were obtained. SD = standard deviation.

To further analyse the impact of systemic intravenous etoposide chemotherapy on the etoposide CSF concentration, the main study group was compared with a second experimental group that did not receive intraventricular etoposide but did receive a CIV etoposide infusion. Figure 2 shows the mean CSF peak and trough concentrations in the main study group (15 courses, 5 patients) following intraventricular etoposide administration on 5 consecutive days and the mean CSF concentration in the second group (5 courses, 2 patients) following a CIV etoposide infusion of 400 mg m$^{-2}$ over 96 hours. Both CSF peak and trough levels were clearly higher after the intraventricular therapy compared to the intravenous etoposide infusion. The etoposide concentrations achieved by systemic infusion were below 0.1 µg ml$^{-1}$.

### DISCUSSION

In patients with relapsed metastatic brain tumours, the multifocality and the spread of tumour cells throughout the CSF require chemotherapeutic regimens aimed at prolonging survival and providing a possible cure. Besides the intensification of chemotherapy by drug escalation in high-dose regimens, combination therapy consisting of systemic chemotherapy and intra-CSF therapy might be a potentially useful approach to achieving cytotoxic drug concentrations in tumour tissue, brain parenchyma and CSF.

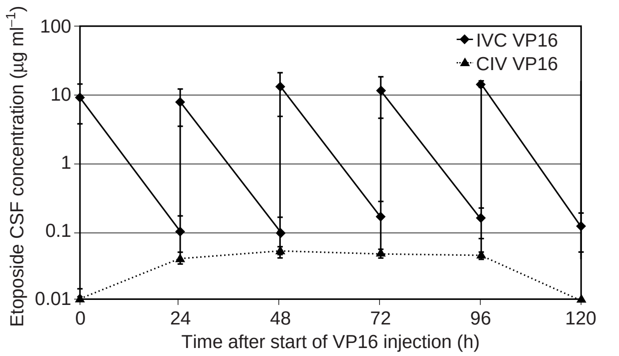

**Figure 2** CSF concentrations (mean ± standard deviation) following intraventricular (IVC) etoposide administration (0.5 mg per day) on 5 consecutive days in the main study group (◆; peak and trough levels, 15 courses in 5 patients) compared with CSF concentration following continuous intravenous infusion (CIV, 400 mg m$^{-2}$ over 96 hours) in the second experimental group (▲; trough and steady state levels, 5 courses in 2 patients)

© 2001 Cancer Research Campaign

**Table 3** Pharmacokinetic parameter in the CSF (mean ± SD) on the first day of intraventricular administration of etoposide (0.5 mg q 24 h, days 1–5) dependent on concurrent systemic chemotherapy with etoposide (25 mg $m^{-2}$ per day, orally, days 1–21)

| Parameter | Without systemic etoposide (*n* = 7) | Concurrent systemic etoposide* (*n* = 4) | All courses (*n* = 11) |
|---|---|---|---|
| AUC (µg h $ml^{-1}$) | 21.90 ± 10.30 | 30.44 ± 5.04 | 25.01 ± 9.48 |
| $t_{1/2\,\alpha}$ (h) | 1.15 ± 0.41 | 1.13 ± 0.21 | 1.14 ± 0.34 |
| $t_{1/2\,\beta}$ (h) | 7.65 ± 1.24 | 6.98 ± 1.06 | 7.41 ± 1.17 |
| $Cl_{tot}$ (ml $min^{-1}$) | 0.56 ± 0.48 | 0.28 ± 0.05 | 0.46 ± 0.40 |
| $V_{ss}$ (l) | 0.20 ± 0.18 | 0.08 ± 0.04 | 0.16 ± 0.15 |

SD = standard deviation. *Oral etoposide was given 12 to 16 hours before intraventricular administration.

The systemic, i.e. intravenous or oral, chemotherapy should provide an effective control of bulk disease and prevent systemic tumour dissemination. The goals of intra-CSF therapy are to treat clinical and subclinical leptomeningeal and pachymeningeal deposits and intraventricular and intraspinal non-parenchymatous metastases as well as floating tumour cells in the CSF and to prevent further seeding into the leptomeninges (Grossman and Krabak, 1999).

In this study, we report on the feasibility of intraventricular administration of etoposide in heavily pretreated patients with recurrent metastatic brain tumours who suffered a first to third relapse. The efficacy of the intraventricular therapy alone could not be assessed because all patients received additional systemic chemotherapy concurrently or underwent repeated irradiation. However, 6 out of 11 patients who had been previously treated with regimens containing etoposide as short-term intravenous infusions responded to the combined therapy in our study. This fact and the response in patients with spinal and/or ventricular metastases and/or with a positive CSF cytology suggest the achievement of only suboptimal tumour cell drug concentrations in their previous treatment (with the dose and schedule of etoposide used) rather than the existence of chemoresistance. However, the observed mixed response in 2 patients, with the regression of spinal metastases and the simultaneous development of new cerebral parenchymatous metastases, suggests insufficient drug penetration into the parenchyma after the intraventricular and systemic chemotherapy in these patients. The results of the animal study by Savaraj et al (1992) support this assumption. Following intracisternal etoposide administration in dogs, they detected high CSF levels but tissue levels that fell exponentially with the distance from the injection site. Thus, the response of parenchymatous metastases or of local tumour recurrence cannot serve to evaluate the effectiveness of intraventricular therapy with etoposide. It is difficult to score response in these metastatic and heavily pretreated patients by other means because of difficulties in quantifying accurately the tumour burden on the surface of the brain and of the spinal cord, in the CSF and in the meninges. However, in a future phase II trial of intraventricularly administered etoposide the tumour response documented by (i) cerebral and spinal MRI scans to evaluate meningeal and intracavitary metastases and (ii) cytology of the ventricular and lumbar CSF to evaluate floating tumour cells should serve as a prospective end-point of the study.

In accordance with previous studies, the intraventricular administration of etoposide at a dose of 0.5 mg on 5 consecutive days at a 2- to 5-week interval is well tolerated and has low toxicity (Van der Gaast et al, 1992; Slavc et al, 2000). The catheter-associated complication rate was similar to that reported by Chamberlain et al (1997) in a larger cohort of 120 patients using Ommaya reservoirs and other intraventricular catheter systems for intraventricular chemotherapy. In our study the intraventricular administration of etoposide via an Ommaya or Rickham reservoir proved to be easy and safe. 1 of the 14 study patients sustained a severe complication with a transient coma following the second intraventricular etoposide administration. Though etoposide toxicity could not be excluded, the documented progression of disease was more likely to have been the cause of the coma. Although we observed no acute or chronic neurological side effects apart from transient mild headaches in any other patients, CSF flow disturbances or CSF space obstructions might theoretically result in extremely high levels of etoposide itself and of its solvents. This might produce reversible or permanent damage to the brain tissue, as was reported in an animal study of intracisternal etoposide administration (Savaraj et al, 1992). To guarantee a rapid dilution of etoposide and its solvents in the CSF and adequate distribution of the drug in the subarachnoid space, as well as to prevent any leakage of the drug into the epidural or subdural space, the intraventricular route was chosen for etoposide administration. In addition, this route makes administration easier and more tolerable for patients than the intralumbar route. To evaluate the CSF flow before starting the intra-CSF therapy, radionuclide ventriculography with 111-Indium-DTPA is recommended in the future for all patients with suspected CSF flow disturbances in the MRI (Chamberlain et al, 1999).

Our pharmacokinetic analysis revealed significant cytotoxic drug concentrations of etoposide in the CSF following intraventricular administration, compared to low levels achieved by exclusively continuous intravenous administration. In this study the CSF levels in patients receiving etoposide only orally were not determined. Taking into account that CSF etoposide levels did not differ between patients with or without systemic oral etoposide administration concurrently with the intraventricular application, we assume that the CSF levels achieved after exclusively oral etoposide administration are low. Etoposide is a lipophilic drug that shows high plasma protein binding and therefore low penetration into the CSF. Assuming that an etoposide level of 0.1 µg $ml^{-1}$ is the minimal cytotoxic level in the CSF (Henwood and Bragden, 1990), cytotoxic drug concentrations may be achieved only by high-dose intravenous infusions (Hande et al, 1984; Postmus et al, 1984) or by intraventricular administration. The concentrations achieved after intraventricular administration of 0.5 mg etoposide exceed by 2- to 10-fold those resulting from high-dose intravenous administration and may provide increased cytotoxic activity against tumour cell deposits in the CSF space. However, cytotoxic drug levels might be obtained by CIV infusions of etoposide or

 © 2001 Cancer Research Campaign oral etoposide, when the blood–CSF barrier is altered and more permeable, i.e. by tumour cell infiltration of the brain or the leptomeninges (Kiya et al, 1992; Relling et al, 1996).

Our study is the first detailed report of pharmacokinetic parameters of etoposide following intraventricular administration in humans. After rapid distribution, the etoposide concentration in the CSF declines in a biexponential pattern. Because of the low protein concentration of ventricular CSF (< 50 mg $dl^{-1}$), the protein binding of etoposide in the CSF is low and this lipophilic drug is rapidly cleared from the CSF, e.g. by CSF bulk flow. Following an intraventricular administration of 0.5 mg etoposide for 5 consecutive days, no drug accumulation was observed. The high interindividual variability of the pharmacokinetic data (AUC, clearance, volume of distribution) indicates that the CSF concentrations might be influenced by individual factors such as CSF volume, CSF flow disturbances or obstructions, increased CSF pressure, increased permeability of the CSF–brain barrier by tumour cell infiltration, and cellular uptake and metabolism.

Our preliminary data suggest that repeated intraventricular etoposide administration is only minimally toxic and is well tolerated. It seems to have a cytotoxic effect on metastatic medulloblastoma and should be tested in phase II trials of this or other brain tumours and neoplastic meningeosis. Further studies are needed to identify patient characteristics affecting the CSF pharmacodynamics and pharmacokinetics of etoposide, in order to optimize the dose and schedule of its intraventricular administration.

### ACKNOWLEDGEMENTS

We thank the following colleagues who enrolled patients in this study: J. D. Beck (Erlangen), U. Goebel (Duesseldorf), S. Haase (Kiel), N. Jorch (Bielefeld), Y. D. Ko (Bonn), R. Mertens (Aachen), O. Peters (Regensburg), R. Straeter (Muenster), S. Voelpel (Krefeld) and T. Wiesel (Datteln).

### REFERENCES

Berg SL, Balis FM, Zimm S, Murphy RF, Holcenberg J, Sato J, Reaman G, Steinherz P, Gillespie A and Doherty K (1992) Phase I/II trial and pharmacokinetics of intrathecal diaziquone in refractory meningeal malignancies. *J Clin Oncol* **10**: 143–148

Bergman I, Pohl CR, Venkataramanan R, Burckart GJ, Stabin M, Barmada MA, Griffin JA and Cheung NK (1999) Intrathecal administration of an anti-ganglioside antibody results in specific accumulation within meningeal neoplastic xenografts in nude rats. *J Immunother* **22**: 114–123

Blaney SM, Balis FM and Poplack DG (1991a) Current pharmacological treatment approaches to central nervous system leukemia. *Drugs* **41**: 702–716

Blaney SM, Balis FM and Poplack DG (1991b) Pharmacologic approaches to the treatment of meningeal malignancy. *Oncology Huntingt* **5**: 107–116

Blaney SM, Cole DE, Godwin K, Sung C, Poplack DG and Balis FM (1995): Intrathecal administration of topotecan in nonhuman primates. *Cancer Chemother Pharmacol* **36**: 121–124

Blasberg RG, Patlak C and Fenstermacher JD (1975) Intrathecal chemotherapy: brain tissue profiles after ventriculocisternal perfusion. *J Pharmacol Exp Ther* **195**: 73–83

Chamberlain MC, Khatibi S, Kim JC, Howell SB, Chatelut E and Kim S (1993) Treatment of leptomeningeal metastasis with intraventricular administration of depot cytarabine (DTC 101). A phase I study. *Arch Neurol* **50**: 261–264

Chamberlain MC, Kormanik PA and Barba D (1997) Complications associated with intraventricular chemotherapy in patients with leptomeningeal metastases. *J Neurosurg* **87**: 694–699

Chamberlain MC, Kormanik P, Jaeckle KA and Glantz M (1999) 111-Indium-diethylenetriamine pentaacetic acid CSF flow studies predict distribution of intrathecally administered chemotherapy and outcome in patients with leptomeningeal metastases. *Neurology* **52**: 216–217

Dunkel IJ, Boyett JM, Yates A, Rosenblum M, Garvin JH, Bostrom BC, Goldman S, Sender LS, Gardner SL, Li H, Allen JC and Finlay JL (1998) High-dose carboplatin, thiotepa, and etoposide with autologous stem-cell rescue for patients with recurrent medulloblastoma. Children's Cancer Group. *J Clin Oncol* **16**: 222–228

Farina P, Marzillo G and D'Incalci M (1981) High-performance liquid chromatography determination of 4′-demethyl-epipodophyllotoxin-9-(4,6-O-ethylidene-beta-D-glucopyraniso-dase) (VP16-213) in human plasma. *J Chromatogr* **222**: 141–145

Grossman SA and Krabak MJ (1999) Leptomeningeal carcinomatosis. *Cancer Treat Rev* **25**: 103–119

Hande KR, Wedlund PJ, Noone RM, Wilkinson GR, Greco FA and Wolff SN (1984) Pharmacokinetics of high-dose etoposide (VP-16-213) administered to cancer patients. *Cancer Res* **44**: 379–382

Henwood JM and Brogden RN (1990) Etoposide. A review of its pharmacodynamic and pharmacokinetic properties, and therapeutic potential in combination chemotherapy of cancer. *Drugs* **39**: 438–490

Kiya K, Uozumi T, Ogasawara H, Sugiyama K, Hotta T, Mikami T and Kurisu K (1992) Penetration of etoposide into human malignant brain tumors after intravenous and oral administration. *Cancer Chemother Pharmacol* **29**: 339–342

Kleihues P, Burger PC and Scheithauer BW (1993) The new WHO classification of brain tumours. *Brain Pathol* **3**: 255–268

Kuehl J, Berthold F, Bode U, Bucsky P, Graf N, Grel A, Maass E, Bamberg M, Kaatsch P, Kleihus P, Rating D, Riehm H and Soerenson N (1993) Preradiation chemotherapy of children with poor prognosis medulloblastoma. *Am J Pediatr Hematol Oncol* **15** (suppl A): 67–71

Kuehl J, Mueller HL, Berthold F, Kortmann RD, Deinlein F, Maass E, Graf N, Gnekow A, Scheurlen W, Goebel U, Wolff JE, Bamberg M, Kaatsch P, Kleihues P, Rating D, Soerensen N and Wiestler OD (1998) Preradiation chemotherapy of children and young adults with malignant brain tumors: results of the German pilot trial HIT '88/' 89. *Klin Paediatr* **210**: 227–233

Nakagawa H, Yamada M, Fukushima M and Ikenaka K (1999) Intrathecal 5-fluoro-2′-deoxyuridine (FdUrd) for the treatment of solid tumor neoplastic meningitis: an in vivo study. *Cancer Chemother Pharmacol* **43**: 247–256

Perek D, Perek-Polnik M and Drogosiewicz M (1999) Pattern of relapse and outcome following recurrence of disease in 105 patients with MB/PNET. *Med Pediatr Oncol* **33**: 290

Postmus PE, Holthuis JJ, Haaxma RH, Mulder NH, Vencken LM, van Oort WJ, Sleijfer DT and Sluiter HJ (1984) Penetration of VP 16–213 into cerebrospinal fluid after high-dose intravenous administration. *J Clin Oncol* **2**: 215–220

Relling MV, Mahmoud HH, Pui CH, Sandlund JT, Rivera GK, Ribeiro RC, Crist WM and Evans WE (1996) Etoposide achieves potentially cytotoxic concentrations in CSF of children with acute lymphoblastic leukemia. *J Clin Oncol* **14**: 399–404

Rubinstein LJ, Herman MM, Long TF and Wilbur JR (1975) Disseminated necrotizing leukoencephalopathy: a complication of treated central nervous system leukemia and lymphoma. *Cancer* **35**: 291–305

Sampson JH, Archer GE, Villavicencio AT, McLendon RE, Friedman AH, Bishop WR, Bigner DD and Friedman HS (1999) Treatment of neoplastic meningitis with intrathecal temozolomide. *Clin Cancer Res* **5**: 1183–1188

Savaraj N, Feun LG, Lu K, Gray K, Wang C and Loo TL (1992) Pharmacology of intrathecal VP-16-213 in dogs. *J Neurooncol* **13**: 211–215

Schmandt S and Kuehl J (1998) Chemotherapy as prophylaxis and treatment of meningeosis in children less than 3 years of age with medulloblastoma. *J Neurooncol* **38**: 187–192

Slavc I, Schuller E, Czech T, Hainfellner JA, Seidl R and Dieckmann K (1998) Intrathecal mafosfamide therapy for pediatric brain tumors with meningeal dissemination. *J Neurooncol* **38**: 213–218

Slavc I, Gulesserian T, Flager J, Guenes M, Schuller E, Czech T and Dieckmann K (2000) Feasibility of long-term intraventricular therapy with mafosfamide (n=22) and VP-16 (n=7) in 22 heavily pretreated and irradiated pediatric brain tumor patients. *Neuro-Oncology* **2**: S92

Stremetzne S, Jaehde U and Schunack W (1997) Determination of the cytotoxic catechol metabolite of etoposide (3′O-demethyletoposide) in human plasma by high-performance liquid chromatography. *J Chromatogr B Biomed Sci Appl* **703**: 209–215

Tomlinson FH, Lihou MG and Smith PJ (1991) Comparison of in vitro activity of epipodophyllotoxins with other chemotherapeutic agents in human medulloblastomas. *Br J Cancer* **64**: 1051–1059

Van der Gaast A, Sonneveld P, Mans DR and Splinter TA (1992) Intrathecal administration of etoposide in the treatment of malignant meningitis: feasibility and pharmacokinetic data. *Cancer Chemother Pharmacol* **29**: 335–337

World Health Organization (1979) WHO Handbook for reporting results of cancer treatment. WHO Offset Publication **48**: 1–41

*© 2001 Cancer Research Campaign* 
